# Effects of a two-week modified ketogenic diet on circulating lipoprotein subclasses, GDF15, and FGF21 in obese adults

**DOI:** 10.1186/s12967-025-07251-2

**Published:** 2025-11-07

**Authors:** Nana Zhang, Na Liu, Guoxia Zhao, Juan Yan, Pinghua Zhang, Xiaomiao Li, Jie Zhou

**Affiliations:** Department of Endocrinology and Metabolism, Xijing Hospital of The Fourth Military Medical University, Xi’an, Shaanxi 710032 China

**Keywords:** Ketogenic diet, Growth differentiation factor 15, Fibroblast growth factor 21, Obesity, Lipoprotein subclasses

## Abstract

**Background:**

As key metabolic regulators, the roles of GDF15 and FGF21 in mediating the effects of modified ketogenic diet (MKD) on weight loss and lipoprotein remodeling in obese patients require further investigation.

**Patients and methods:**

This study enrolled 30 metabolically healthy obese participants (BMI ≥ 28 kg/m²) for a 2-week MKD intervention. Using a self-controlled pre-post design, we performed measurements including body composition analysis, fasting serum GDF15 and FGF21 levels measurement, and serum lipoprotein subclass quantification at both baseline and post-intervention time points.

**Results:**

Following a 2-week MKD intervention, participants exhibited statistically significant reductions in body weight (96.14 ± 27.23 kg vs. 91.63 ± 26.47 kg; Δ4.8%, *P* < 0.001) and BMI (33.99 ± 6.08 kg/m^2^ vs. 32.41 ± 5.95 kg/m^2^; Δ4.7%, *P* < 0.001). Body fat parameters significantly improved, with body fat mass (BFM) and visceral fat area (VFA) decreasing by > 5%. Meanwhile, lean mass indices (SMM, SLM, FFM) remained stable (change < 3%). Serum biomarker analysis revealed that GDF15 levels increased significantly by 5.76% (*P* = 0.0377), whereas FGF21 levels decreased markedly by 51.91% (*P* = 0.0001). The apolipoprotein B/A1 ratio (t = 5.381, *P* < 0.001) and the LDL-c/HDL-c ratio (t = 5.095, *P* < 0.001) increased significantly. Furthermore, larger HDL-c subfractions (H1FC/H2FC) showed an upward trend, while smaller HDL-c subfractions (H3FC/H4FC) exhibited a downward trend. Among these changes, H2FC levels demonstrated the most pronounced elevation (t = 6.119, *P* < 0.001).

**Conclusion:**

The short-term MKD intervention significantly improved adiposity metrics while elevating GDF15 and reducing FGF21 levels. These rapid metabolic adaptations induced potentially beneficial remodeling of HDL-c subclasses, highlighting novel effects beyond conventional lipid ratios.

**Trial registration:**

ChiCTR, ChiCTR2300071823. Registered 25 May 2023 - Prospective Registered, https://www.chictr.org.cn/bin/project/edit?pid=198176.

## Introduction

Obesity has become a major global public health challenge. According to the World Obesity Atlas 2025, the number of obese adults worldwide is projected to surge from 524 million in 2010 to 1.13 billion by 2030, an increase of over 115%. In China, the number of overweight/obese adults is expected to reach 515 million [1]. Obesity not only impacts physical appearance but is also strongly associated with diabetes, hypertension, cardiovascular diseases, and other health conditions [2]. Approximately, 1.6 million premature deaths annually are attributed to obesity, with 55% of type 2 diabetes-related premature deaths linked to obesity [3]. Therefore, it is imperative to explore weight management strategies that demonstrate efficacy, feasibility, and optimal patient adherence.

The classic ketogenic diet (KD), as a low-carbohydrate and high-fat dietary regimen, has been demonstrated by numerous studies to be effective for weight reduction and improvement of metabolic parameters [4–6]. Although, KD has demonstrated therapeutic potential for obesity, it has not been endorsed as a first-line treatment in clinical guidelines. The primary limitations include: poor long-term adherence in Chinese populations accustomed to traditional carbohydrate-rich diets (providing over 60% of energy intake), and significant reduction in cardioprotective foods such as whole grains, legumes, and dark green leafy vegetables. To address these critical clinical challenges, our study has innovatively developed a Modified Ketogenic Diet (MKD) better adapted to Chinese dietary patterns. This protocol features an optimized macronutrient distribution: 20% carbohydrates, 40% protein, and 40% fat, ensuring concurrent ketogenic efficacy and cultural compatibility.

Growth differentiation factor 15 (GDF15), a unique member of the transforming growth factor-β (TGF-β) superfamily, exhibits distinctive biological characteristics. As a pleiotropic cytokine, GDF15 plays a crucial role in stress responses following cellular injury. Its most prominent biological function involves regulating energy metabolism by suppressing appetite and reducing food intake, earning it the scientific designation of “anorexigenic hormone” [7, 8]. Recently studies have demonstrated that the signaling pathway formed by GDF15 and its specific receptor GDNF-family receptor α-like (GFRAL) plays a pivotal role in various metabolic disorders and pathological processes [9]. Currently, this signaling system has been established as an important biomarker for multiple diseases including obesity, type 2 diabetes mellitus, malignant tumors, and metabolic-associated fatty liver disease (MAFLD), providing new research directions for the diagnosis and treatment of related conditions [10, 11].

Fibroblast growth factor 21 (FGF21), a key member of the FGF19 subfamily, is predominantly expressed in liver tissue. It exerts its metabolic regulatory functions by targeting multiple organs including adipose tissue, liver, and pancreas to modulate glucose and lipid homeostasis [12, 13]. Research has demonstrated that FGF21 possesses multifaceted metabolic effects, including body weight reduction, improvement of insulin sensitivity, and reversal of hepatic steatosis, highlighting its significant physiological roles [14, 15].

Current evidence indicates that the anti-obesity effects of the classic KD are primarily mediated through two distinct pathways: GDF15-induced appetite suppression (reducing energy intake) and FGF21-enhanced energy expenditure (increasing metabolic activity) [16]. To investigate whether the MKD exerts its weight-loss effects, we conducted a 2-week MKD intervention in obese subjects, aiming to systematically evaluate its efficacy for weight reduction and identify potential circulating biomarkers related to its metabolic effects.

## Materials and methods

### Study participants

Between September 2023 and March 2024, we prospectively enrolled 35 metabolically healthy obese individuals (BMI ≥ 28 kg/m^2^) from the Endocrinology and Metabolism Clinic at the Xijing Hospital of The Fourth Military Medical University. All eligible participants underwent standardized baseline health assessments prior to study participation. Exclusion Criteria: (a) Secondary obesity (Cushing’s syndrome, hypothyroidism, growth hormone-secreting tumors, etc.); (b)Current use of obesity-inducing medications (e.g., corticosteroids, psychotropic drugs); (c)Conditions including pernicious anemia, malignancies, acute/chronic infections, severe hepatic/renal dysfunction, severe cardiopulmonary diseases, or cerebrovascular disorders; (d) Pregnancy or lactation status; (e) Diagnosis of type 2 diabetes mellitus; (f) Current use of antidiabetic drugs or lipid-lowering medications. During the study period, three participants withdrew and two others had insufficient key parameter data. Consequently, the final analysis included complete datasets from 30 participants.

The study protocol received ethical approval from the Medical Ethics Committee of the Xijing Hospital of Fourth Military Medical University (Approval No. KY20232166) and was prospectively registered with the Chinese Clinical Trial Registry (Registration No. ChiCTR 2300071823). All participants provided written informed consent. The investigation was conducted in full accordance with the ethical principles outlined in the Declaration of Helsinki.

### Study protocol

One week prior to the study initiation, all participants were instructed to maintain their habitual dietary intake. After enrollment, participants received individualized weight-loss interventions and were required to collect first-void morning urine samples daily for urinary ketone assessment. Fasting serum samples and anthropometric measurements were obtained at two time points: baseline (pre-intervention) and 2-week follow-up (post-intervention). All serum samples were immediately aliquoted and stored at -80 °C until further analysis.

### Diet interventions

This self-controlled, paired study implemented a comprehensive 2-week weight management program incorporating dietary, exercise, and psychological interventions. Participants received personalized KD plans (20% carbs, 40% protein, 40% fat) with daily caloric intake calculated as 70% of actual weight (kg) × (20–25 kcal). Prior to the intervention, each participant received a personalized meal plan from clinical nutritionists. Adherence was supported through daily remote monitoring via WeChat, which included morning urine ketone self-testing (values of +- to + + confirmed ketosis), submission of daily photo logs for meals and hydration, and provision of real-time feedback and psychological support to ensure protocol compliance. To minimize variations in energy expenditure, all participants were instructed to maintain their habitual levels of physical activity for the duration of the study. The primary endpoints included changes in body weight, GDF15, FGF21 levels, and serum metabolomic profiles after the 2-week intervention period.

### Data collection

#### Body composition analysis

Body composition assessment was conducted using the InBody 770 bioelectrical impedance analyzer (Biospace Inc., Seoul, Korea) according to manufacturer’s protocols. Prior to assessment, participants were required to remove outer clothing and metallic items (e.g., watches and necklaces), stand barefoot on the footplate electrodes, and grasp the hand electrodes with both hands. The recorded parameters included height, weight, BMI, waist-to-hip ratio, fat mass, skeletal muscle mass, and visceral fat area, which were collected at both baseline and post-intervention.

#### Serum biomarker analysis

Serum biomarkers were quantified using ELISA assays: GDF15 was measured with Human GDF15 Quantikine ELISA Kit (R&D Systems, USA; Cat# DY957; range: 31.2–2000 pg/mL; sensitivity: <10 pg/mL), and FGF21 with Human FGF21 Quantikine ELISA Kit (Cat# DF2100; range: 15.6–1000 pg/mL; sensitivity: <5 pg/mL). All samples were analyzed in duplicate (inter-assay CV < 12%) with kit standards and internal controls. Plate readers were calibrated per manufacturer’s protocols, and out-of-range samples were re-tested after appropriate dilution. Paired blood samples were collected at baseline and post-intervention for biomarker analysis.

#### Serum lipoprotein subclasses analysis

Serum lipoprotein subclasses analysis was conducted using the Bruker AVANCE IVDr NMR metabolomics profiling system for comprehensive ¹H-NMR-based metabolic profiling. All procedures were performed in strict accordance with the IVDr Standard Operating Procedures (SOP) to quantify lipoprotein subclasses. Nuclear magnetic resonance (NMR) lipoprotein subfraction quantification includes subtypes of very low-density lipoprotein cholesterol (VLDL-c), low-density lipoprotein cholesterol (LDL-c), intermediate-density lipoprotein cholesterol (IDL-c) and high-density lipoprotein cholesterol (HDL-c), totaling 114 lipoprotein particles. Paired blood samples were collected at baseline and post-intervention for metabolomics analysis.

### Statistical analysis

All statistical analyses were performed using SPSS software (version 26.0; IBM Corporation, Armonk, NY, USA) and MetaboAnalyst platform (version 6.0; McGill University, Montreal, QC, Canada; https://www.metaboanalyst.ca, accessed on 12 May 2025). Paired t-test analysis was performed to assess changes in body composition parameters pre- and post-MKD intervention. Non-normally distributed variables underwent logarithmic transformation. Candidate biomarkers were identified through comprehensive bioinformatics analysis. Receiver operating characteristic (ROC) curves were constructed using orthogonal partial least squares-discriminant analysis (OPLS-DA) methodology for comprehensive evaluation and screening of potential biomarkers. The OPLS-DA score chart was used to find different lipoprotein subclasses through the obtained VIP values. We used OPLS-DA to calculate the VIP value, which was analyzed with VIP > 1 and a t-test (*P* < 0.05), to screen the differential lipoprotein subclasses between groups. Furthermore, we evaluated the correlation between the concentration of GDF15, FGF21 and body composition, as well as lipid subclasses.

## Results

### Effects of a 2-week MKD on body composition in obese adults

**Fig. 1:**
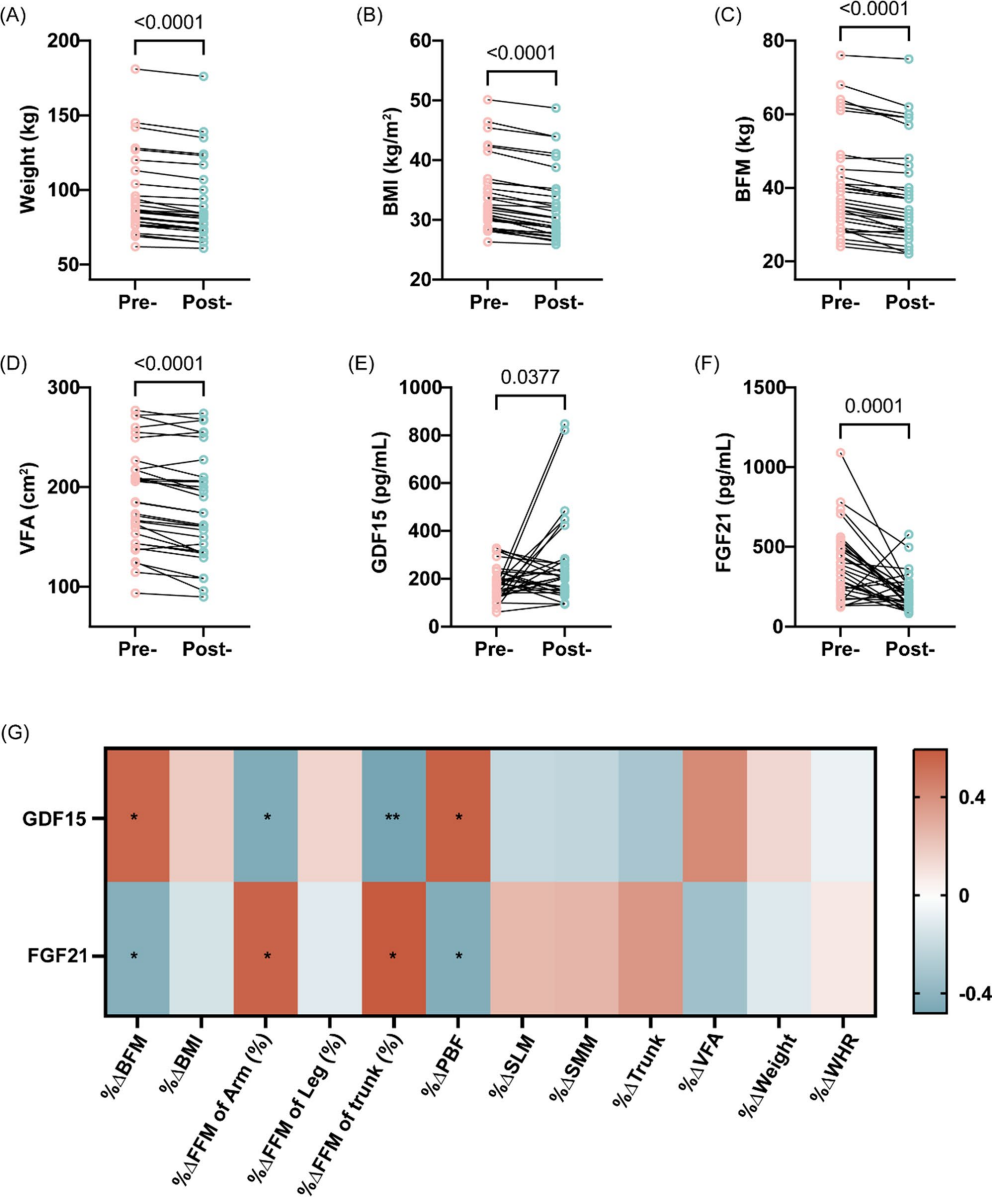
Effects of a 2-week MKD on body composition and serum biomarkers in obese adults. (**A**-**F**) Changes in Weight (**A**), BMI (**B**), BFM (**C**), VFA (**D**), GDF15 (**E**) and FGF21 (**F**) after 2-week MKD. (**G**) Correlation matrix of GDF15/FGF21 with body composition (blue/red: positive/negative correlation). Abbreviation: BFM: Body Fat Mass; BMI: Body Mass Index; VFA: Visceral Fat Area. ^*^P < 0.05, ^**^P < 0.01, ^***^P < 0.001

To assess the impact of a 2-week MKD on body composition, all participants adhered to the diet and achieved ketosis, as quantitatively verified via ¹H-NMR metabolomics (Table 1). As shown in Table 1; Fig. 1, participants exhibited statistically significant reductions in both body weight (96.14 ± 27.23 kg vs. 91.63 ± 26.47 kg; Δ4.8%, *P* < 0.001) and BMI (33.99 ± 6.08 kg/m^2^ vs. 32.41 ± 5.95 kg/m^2^; Δ4.7%, *P* < 0.001) following the 2-week MKD intervention. Notably, patients exhibited significant reductions in body fat parameters, with BFM and VFA decreasing > 5% and PBF declining by 2.71%. Conversely,, lean mass indices (SMM, SLM, FFM) remained stable (change < 3%), indicating that the MKD promoted fat loss while effectively preserving lean mass.

**Table 1:** Effects of a 2-Week MKD on body composition and ketone body in obese adults

	Pre-	Post-	Change (%)	t	*P*
Age (years)	31.00 ± 8.56				
Sex (Female%)	22 (71%)				
Weight (kg)	96.14 ± 27.23	91.63 ± 26.47	4.76	12.743	< 0.001
BMI (kg/m^2^)	33.99 ± 6.08	32.41 ± 5.95	4.71	13.681	< 0.001
WHR	0.97 ± 0.08	0.97 ± 0.08	0.01	-0.245	0.808
BFM (kg)	41.10 ± 13.89	38.28 ± 13.73	7.32	9.020	< 0.001
VFA (cm^2^)	188.00 ± 50.45	178.97 ± 53.63	5.45	5.236	< 0.001
PBF (%)	42.40 ± 5.42	41.29 ± 5.70	2.71	4.065	< 0.001
SLM (kg)	51.85 ± 14.38	50.25 ± 13.69	2.95	5.826	< 0.001
SMM (kg)	30.57 ± 9.04	29.64 ± 8.71	2.97	5.477	< 0.001
FFM (kg)	55.04 ± 15.21	53.35 ± 14.49	2.93	5.856	< 0.001
FFM of trunk (%)	101.95 ± 5.67	101.21 ± 5.58	0.70	1.927	0.064
FFM of Arm (%)	17.25 ± 4.66	16.66 ± 4.39	1.69	6.266	< 0.001
FFM of Leg (%)	201.83 ± 10.87	198.93 ± 9.70	1.38	3.167	0.005
Acetoacetate	0.11 ± 0.05	0.12 ± 0.05	8.52	0.825	0.416
β-hydroxybutyrate	0.01 ± 0.04	0.04 ± 0.08	393.51	2.060	0.048
Acetone	0.06 ± 0.11	0.16 ± 0.20	164.50	2.142	0.041

Abbreviation: BFM: Body Fat Mass; BMI: Body Mass Index; FFM: Fat Free Mass; PBF: Percent Body Fat; SLM: Soft Lean Mass; SMM: Skeletal Muscle Mass; VFA: Visceral Fat Area; WHR: Waist-Hip Ratio

### Effects of a 2-week MKD on serum GDF15 and FGF21 concentrations in obese adults

We next examined whether MKD modulates key serum biomarkers in obesity. As shown in Fig. 1, serum biomarker GDF15 levels increased significantly by 5.76% (*P* = 0.0377), whereas FGF21 levels decreased markedly by 51.91% (*P* = 0.0001) after the 2-week MKD intervention (Fig. 1E-F). Using body composition change-to-baseline ratios, we further assessed their correlations with baseline levels and intervention-induced changes in GDF15 and FGF21. Notably, baseline circulating GDF15 levels showed significant positive correlations with reductions in body fat indices (%ΔBFM, %ΔPBF; *r* = 0.43 to 0.45, *P* < 0.05), while demonstrating inverse associations with lean mass preservation (%ΔArm, %ΔTrunk; *r*=-0.48 to -0.45, *P* < 0.05). Conversely, baseline FGF21 concentrations exhibited negative correlations with body fat reduction (%ΔBFM, %ΔPBF; *r*=-0.44 to -0.43, *P* < 0.05) but positive correlations with lean mass changes (%ΔArm, %ΔTrunk; *r* = 0.44 to 0.47, *P* < 0.05). These distinct correlation patterns suggest that the magnitude of weight loss during MKD intervention is positively associated with baseline GDF15 levels and negatively associated with FGF21 levels (Fig. 1G).

### Effects of a 2-week MKD Intervention on lipoprotein subclass profiles

**Fig. 2:**
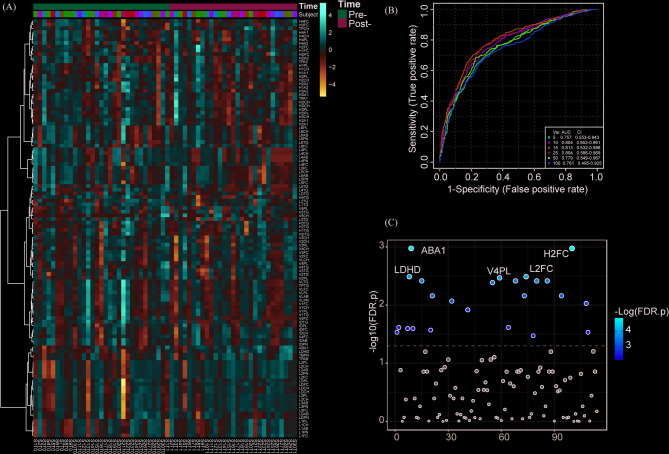
Changes in lipoprotein subclasses following a 2-week MKD intervention. (**A**) Heatmap displaying temporal changes in lipoprotein subclasses during the 2-week MKD intervention (pre- and post-intervention). (**B**) Receiver operating characteristic (ROC) curve analysis (AUC values shown) of plasma lipoprotein subclass profiles comparing pre- and post-MKD intervention states. (**C**) Orthogonal partial least squares-discriminant analysis (orthogonal partial least squares discriminant analysis, OPLS-DA) of lipoprotein fractions and subfractions reveals distinct clustering between pre- and post-MKD intervention groups

To characterize the diet-induced lipoprotein remodeling, we analyzed subclass abundances pre- and post-intervention (Fig. 2A). ROC analysis (AUC = 0.779, Fig. 2B) indicated significant modifications in plasma lipoprotein profiles following the 2-week intervention. Principal component analysis (PCA) revealed distinct clustering between pre- and post-intervention states (Fig. 2C). Notably, 22 lipoprotein subclasses exhibited significant alterations post-MKD (VIP > 1, *P* < 0.05), with the top 15 most substantially changed lipoprotein subclasses highlighted in Fig. 2C. These data demonstrate that short-term MKD induces substantial lipoprotein redistribution.

### Association between circulating GDF15/FGF21 levels and lipoprotein subclass profiles in obese adults

**Fig. 3:**
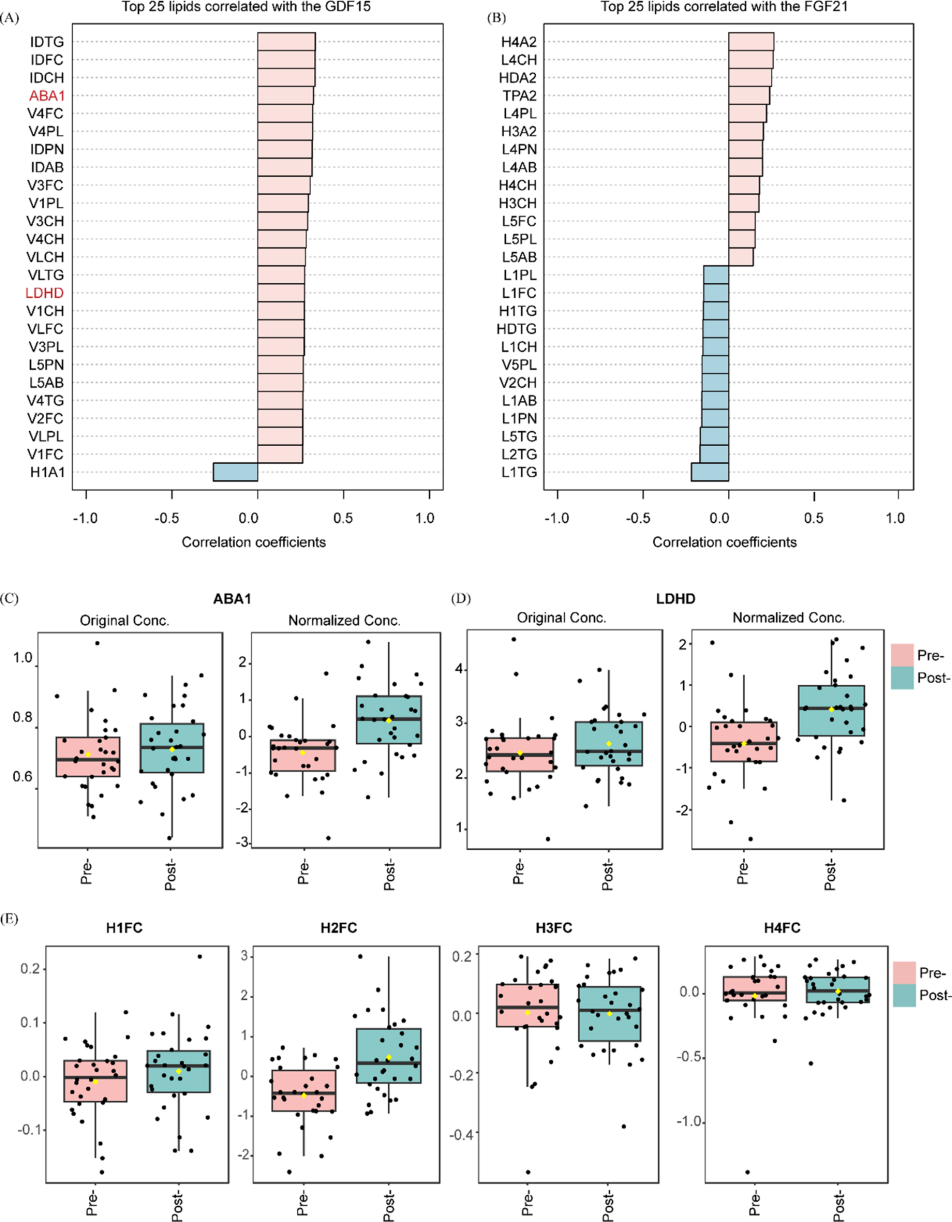
Associations between circulating GDF15/FGF21 levels and lipoprotein subclasses. (**A**-**B**) Correlations of GDF15 and FGF21 with lipoprotein subclasses. (**C**-**D**) Dynamic changes in lipoprotein ratios: ApoB/ApoA1 (ABA1) and LDL-c/HDL-c (LDHD). (**E**) Changes in HDL-c subfractions (H1FC, H2FC, H3FC, H4FC) following MKD intervention

Finally, we investigated whether GDF15 and FGF21 levels correlate with specific lipoprotein changes. Figure 3A and B demonstrate the associations between GDF15/FGF21 and lipoprotein subclass after adjustment for sex, age, and time. The ApoB/ApoA1 (ABA1) ratio (t = 5.381, *P* < 0.001) and LDL-c/HDL-c(LDHD) ratio (t = 5.095, *P* < 0.001) significantly increased during the MKD intervention (Fig. 3C and D). Notably, we observed that larger HDL subfractions (H1FC/H2FC) exhibited an increasing trend following treatment, whereas smaller HDL subfractions (H3FC/H4FC) displayed a decreasing trend. The most pronounced change was the elevation in H2FC levels (t = 6.119, *P* < 0.001) after MKD intervention (Fig. 3E). These findings imply that MKD induces a distinct shift toward larger HDL particles, which correlates with composition of MKD and may reflect altered lipid metabolism.

## Discussion

In the present study, we investigated the effects of short-term MKD intervention on both weight loss and circulating GDF15/FGF21 levels in obese adults. Our results demonstrated that the 2-week MKD intervention induced statistically significant reductions in body weight and BMI, accompanied by an increase in GDF15 and a decrease in FGF21 levels. Further analysis revealed that these changes in biomarkers were closely associated with lipoprotein subclass remodeling, both the ABA1 and LDHD ratios showed significant increases, and HDL-c subclasses exhibited a marked shift toward larger particle sizes (increased H1FC/H2FC and decreased H3FC/H4FC), with the H2FC subclass demonstrating the most prominent elevation.

The KD is a metabolic intervention strategy characterized by low carbohydrate, high fat, and moderate protein intake. Its core mechanism involves shifting the body’s energy metabolism from glucose utilization to fat mobilization and ketone body production. The classical KD employs a strict 4:1 ratio of fat to (carbohydrates + protein), with 90% of calories derived from fat and only 2–3% from carbohydrates [17]. While this extreme ratio has demonstrated significant therapeutic effects in managing metabolic disorders such as obesity, type 2 diabetes, metabolic-associated fatty liver disease, and polycystic ovary syndrome (PCOS), its clinical application faces multiple challenges: the ultra-high fat content results in poor palatability, frequent gastrointestinal discomfort, restriction of cardioprotective foods like whole grains and legumes, and particularly poor long-term adherence among Chinese populations (traditional carbohydrate intake accounts for >60% of energy) [18–21]. To address these limitations, this study innovatively developed a MKD that optimizes macronutrient ratios to 20% carbohydrates, 40% protein, and 40% fat. Clinical research demonstrates that MKD not only preserves the metabolic benefits of traditional KD but also significantly improves its clinical application drawbacks. Just two weeks of MKD intervention achieved a 4.8% weight reduction and 4.7% BMI decrease, with body fat mass and visceral fat area significantly reduced by >5%, while effectively maintaining lean body mass stability (change < 3%). This modified approach not only enhances dietary variety and patient tolerance but also provides a more sustainable metabolic management solution for populations accustomed to high-carbohydrate diets, such as the Chinese population.

The discovery of the GDF15-GFRAL axis in 2017 opened new therapeutic avenues for obesity treatment [22]. This signaling pathway not only suppresses food intake through central mechanisms but also exerts peripheral anti-obesity effects by promoting thermogenesis, delaying gastrointestinal emptying, enhancing lipolysis, and maintaining skeletal muscle energy expenditure [23–26]. A landmark study by Gregory R. Steinberg’s team at McMaster University, published in Nature, demonstrated that the GDF15-GFRAL pathway increases skeletal muscle calcium futile cycling to simultaneously suppress appetite and maintain energy expenditure during caloric restriction, providing a novel solution to overcome the challenge of “weight rebound” in obesity management [27]. Notably, the anti-obesity effects of metformin, a first-line antidiabetic drug, have been shown to be mediated through GDF15. Accumulating evidence indicates that metformin upregulates GDF15 expression in various tissues (including intestine and kidney), which then acts on the central nervous system to suppress appetite and regulate energy metabolism [28–30]. Furthermore, the positive feedback loop between GDF15 and AMPK is essential for complete metformin activation, establishing GDF15’s central role in metformin’s metabolic regulatory network [31]. Complementary studies by Wu JW’s team revealed that KD can activate hepatic PPARγ signaling to upregulate GDF15 transcription, thereby promoting weight loss [17]. Our research further demonstrates that MKD intervention significantly elevates circulating GDF15 levels and produces marked weight reduction. Consistent with previous findings, our study not only confirm the critical role of GDF15 in weight regulation, but also provides a clinically feasible long-term dietary intervention strategy for obesity management.

FGF21, as a key metabolic regulatory hormone, demonstrates a specific response pattern to dietary nutrients in both humans and rodents. Substantial research evidence indicates that the expression and secretion of this hormone are precisely regulated by the carbohydrate-protein balance. Regarding nutrient-sensing mechanisms, carbohydrates have been identified as the most potent stimulators of FGF21.Clinical studies show that both acute glucose loading and chronic high-carbohydrate diets can significantly elevate circulating FGF21 concentrations [32–34]. This phenomenon has been further validated in rodent models, manifesting as synchronized increases in peripheral blood FGF21 levels and hepatic FGF21 mRNA expression [35]. In contrast to carbohydrates, dietary protein exerts bidirectional regulatory effects on FGF21: protein restriction significantly elevates FGF21 levels in both humans and animals [36, 37], while moderate protein supplementation effectively counteracts carbohydrate-induced FGF21 elevation [38]. This regulatory effect is most pronounced under high-carbohydrate/low-protein dietary conditions. In our study, MKD (low-carbohydrate/high-protein) intervention induced a rapid decline in plasma FGF21 levels within a short period, independent of energy intake and body weight changes, strongly confirming FGF21’s specific responsiveness to macronutrient composition. Particularly insightful is the observation that the characteristically elevated fasting FGF21 levels in obese individuals [26] were significantly reduced after MKD intervention. This result not only excludes the interference from metabolic diseases per se but also establishes FGF21’s molecular role as a “nutritional status sensor”. From a mechanistic perspective, FGF21’s rapid response to dietary changes likely stems from its unique regulatory properties: increased protein intake directly suppresses FGF21 expression, while reduced carbohydrate availability removes its primary stimulatory signal. Notably, our results reveal a crucial dissociation from classic KD patterns. While both classic KD and our MKD elevate GDF15, our modified diet produced a distinct reduction in FGF21 - contrasting sharply with the characteristic FGF21 elevation typically observed in classic KD studies. This divergent response suggests that the higher protein content (40%) in our MKD protocol may attenuate the typical ketogenic stimulation of FGF21 through hepatic PPARα activation and low-carbohydrate stress signaling. Our findings systematically elucidate the dynamic regulatory patterns of FGF21 by macronutrient composition, providing novel experimental evidence and theoretical foundations for understanding the intricate interplay between diet, hormones and metabolism.

Characterized by a high fat intake (70–80%), the classic KD reduces body weight while improving triglyceride levels, blood pressure, and blood glucose regulation. Nevertheless, it elevates total cholesterol and low-density lipoprotein cholesterol (LDL-c) levels, which are established independent risk factors for cardiovascular disease [39]. Our findings indicate that the MKD similarly affects lipoprotein metabolism in a complex manner. Consistent with the established effects of ketogenic diets, we observed increases in several atherogenic lipid parameters. More notably, our NMR-based profiling revealed a shift in HDL subclasses toward larger particles (H1FC/H2FC), particularly H2FC-a pattern that contrasts with some reports on classic KD and may represent a distinctive feature of our modified macronutrient composition. This shift stands in contrast to the findings of Montefusco et al., who reported that a carbohydrate-moderate hypocaloric diet reduced total HDL TG content, suggesting a decrease in larger, TG-rich HDL particles [40]. The shift toward larger HDL particles observed with the MKD appears to be attributable to its specific dietary composition rather than energy restriction alone, which is further supported by the rapid induction of ketosis and distinct alterations in lipoprotein profiles. The clinical implications of these simultaneous changes, including both elevated atherogenic lipids and a distinct shift in HDL subclasses, require further exploration. Future studies directly comparing the MKD with isocaloric classic KD will help clarify the diet-specific effects on lipoprotein metabolism and their potential long-term cardiovascular outcomes.

This study demonstrates three key strengths. Firstly, this study offers a comprehensive metabolic evaluation by innovatively assessing both weight-loss efficacy and serum biomarker responses (increased GDF15 and decreased FGF21 levels) during short-term MKD intervention, offering multidimensional insights into its metabolic regulatory mechanisms. Notably, MKD induces potentially beneficial remodeling of lipoprotein subclasses. Secondly, the findings possess significant clinical value. Our data showed that a mere 2-week MKD intervention achieved rapid and significant reductions in body weight/BMI, thereby establishing strong evidence for MKD as an effective short-term intervention for obesity. Of particular interest, the opposing regulatory trends observed in GDF15 (anorexigenic) and FGF21 (metabolic regulator) suggest that MKD may modulate energy homeostasis through distinct signaling pathways. Thirdly, this study adheres to stringent scientific methodology. Standardized biomarker detection methods (particularly NMR spectroscopy-based HDL-c subclass analysis) were employed to ensure reproducibility. Additionally, rigorous adjustment for confounders (age, sex, and time) significantly enhanced the validity of the conclusions.

This study has several limitations. Firstly, it is important to note that our models incorporated adjustments for age and gender. Nevertheless, the generalizability of the findings to broader populations is limited by a relatively small size, a higher proportion of female participants, and a relatively young age range. Secondly, the single-arm design without a control group (e.g., a classic KD group) prevents us from establishing definitive the superiority of the MKD. Our results should be interpreted as exploratory. Thirdly, the short-term MKD intervention precludes assessment of whether the observed metabolic changes, such as weight loss, elevated GDF15, reduced FGF21, and lipoprotein subclass remodeling, represent sustained adaptations. Furthermore, although participants were instructed to maintain their habitual physical activity levels and dietary was remotely monitored, the lack of rigorous control and objective measurement of energy expenditure may have introduced confounding. Finally, while we identified associations between dietary effects and biomarker changes, the lack of experimental validation (e.g., in vitro or animal models) prevents establishment of causal mechanisms. Future studies should incorporate larger cohorts, longer intervention periods, controlled dietary comparisons, and mechanistic investigations through multi-omics approaches or functional experiments to validate these preliminary findings.

## Conclusion

This study demonstrates that a short-term MKD intervention significantly improves adiposity metrics in obese individuals and induces distinct alterations in lipoprotein subclass distribution, accompanied by elevated circulating GDF15 and reduced FGF21 levels. Notably, the observed shift in HDL subclasses occurred concurrently with elevations in atherogenic lipid parameters. Therefore, the potential metabolic significance of HDL remodeling must be interpreted with caution. Subsequent studies should focus on long-term outcomes and explore the molecular pathways associated with GDF15 and FGF21 in the regulation of lipoprotein metabolism.

## Data Availability

The datasets in this study were derived from the Department of Endocrinology at the Xijing Hospital of The Fourth Military Medical University. Due to data security and privacy regulations, these datasets are not publicly available. However, they may be obtained from the corresponding author upon reasonable request and with permission from the originating institution.

## Abbreviations

MKD: Modified Ketogenic Diet
GDF15: Growth Differentiation Factor 15
FGF21: Fibroblast Growth Factor 21
GFRAL: GDNF-family receptor α-like
MAFLD: Metabolic-associated Fatty Liver Disease
BFM: Body Fat Mass
BMI: Body Mass Index
FFM: Fat Free Mass
PBF: Percent Body Fat
SLM: Soft Lean Mass
SMM: Skeletal Muscle Mass
VFA: Visceral Fat Area
WHR: Waist-Hip Ratio
SOP: Standard Operating Procedures
OPLS-DA: Orthogonal Partial Least Squares-discriminant Analysis
VIP: Variable importance in projection
VLDL-c: Very Low-density Lipoprotein cholesterol
LDL-c: Low-density Lipoprotein cholesterol
IDL-c: Intermediate-density Lipoprotein cholesterol
HDL-c: High-density lipoprotein cholesterol
TG: Triglycerides
PCOS: Polycystic Ovarian Syndrome

## References

[1] World Obesity Federation. World obesity atlas 2025. London: World Obesity Federation; 2025.

[2] Aronne LJ, Bramblette S, Huett-Garcia A, Ingelfinger JR, Jastreboff AM, Machineni S, Massie N, Rosen CJ. Weight and Health - Pathophysiology and therapies. N Engl J Med. 2022;387(24):e62. 36516089 10.1056/NEJMp2214423

[3] Pan XF, Wang L, Pan A. Epidemiology and determinants of obesity in China. Lancet Diabetes Endocrinol. 2021;9(6):373–92. 34022156 10.1016/S2213-8587(21)00045-0

[4] Khalid K, Apparow S, Mushaddik IL, Anuar A, Rizvi SAA, Habib A. Effects of ketogenic diet on reproductive hormones in women with polycystic ovary syndrome. J Endocr Soc. 2023;7(10):bvad112. 37693687 10.1210/jendso/bvad112 PMC10484165

[5] Baylie T, Ayelgn T, Tiruneh M, Tesfa KH. Effect of ketogenic diet on obesity and other metabolic disorders: narrative review. Diabetes Metab Syndr Obes. 2024;17:1391–401. 38529169 10.2147/DMSO.S447659 PMC10962461

[6] Dyńka D, Rodzeń Ł, Rodzeń M, Pacholak-Klimas A, Ede G, Sethi S, et al. Ketogenic diets for body weight loss: a comparison with other diets. Nutrients. 2025;17(6):965. 40289934 10.3390/nu17060965 PMC11945412

[7] Zhang J, Sun J, Li J, Xia H. Targeting the GDF15 signalling for obesity treatment: recent advances and emerging challenges. J Cell Mol Med. 2024;28(24):e70251. 39700016 10.1111/jcmm.70251 PMC11657595

[8] Li J, Hu X, Xie Z, Li J, Huang C, Huang Y. Overview of growth differentiation factor 15 (GDF15) in metabolic diseases. Biomed Pharmacother. 2024;176:116809. 38810400 10.1016/j.biopha.2024.116809

[9] Di Santo A, Tarchi L, Villa G, Castellini G, Ricca V, Squecco R, et al. GDF15 analogues acting as GFRAL ligands. ChemMedChem. 2025;20(9):e202400961. 39907315 10.1002/cmdc.202400961 PMC12058240

[10] Breit SN, Brown DA, Tsai VW. The GDF15-GFRAL pathway in health and metabolic disease: friend or foe? Annu Rev Physiol. 2021;83:127–51. 33228454 10.1146/annurev-physiol-022020-045449

[11] Wang D, Day EA, Townsend LK, Djordjevic D, Jørgensen SB, Steinberg GR. GDF15: emerging biology and therapeutic applications for obesity and cardiometabolic disease. Nat Rev Endocrinol. 2021;17(10):592–607. 34381196 10.1038/s41574-021-00529-7

[12] Trusz GJ. Fibroblast growth factor 21. Differentiation. 2024 Sep-Oct;139:100793. 10.1016/j.diff.2024.100793 38991938

[13] Szczepańska E, Gietka-Czernel M. FGF21: A novel regulator of glucose and lipid metabolism and whole-body energy balance. Horm Metab Res. 2022;54(4):203–11. 35413740 10.1055/a-1778-4159

[14] Geng L, Lam KSL, Xu A. The therapeutic potential of FGF21 in metabolic diseases: from bench to clinic. Nat Rev Endocrinol. 2020;16(11):654–67. 32764725 10.1038/s41574-020-0386-0

[15] Tillman EJ, Rolph T. FGF21: an emerging therapeutic target for non-alcoholic steatohepatitis and related metabolic diseases. Front endocrinol (Lausanne). 2020 Dec 14;11:601290. 10.3389/fendo.2020.601290 PMC7767990 33381084

[16] Lu JF, Zhu MQ, Xia B, Zhang NN, Liu XP, Liu H, et al. GDF15 is a major determinant of ketogenic diet-induced weight loss. Cell Metab. 2023;35(12):2165–e21827. 38056430 10.1016/j.cmet.2023.11.003

[17] Charlot A, Zoll J. Beneficial effects of the ketogenic diet in metabolic syndrome: a systematic review. Diabetology. 2022;3(2):292–309.

[18] Dyńka D, Kowalcze K, Charuta A, Paziewska A. The ketogenic diet and cardiovascular diseases. Nutrients. 2023;15(15):3368. 37892389 10.3390/nu15204312 PMC10609625

[19] Watanabe M, Tozzi R, Risi R, Tuccinardi D, Mariani S, Basciani S, et al. Beneficial effects of the ketogenic diet on nonalcoholic fatty liver disease: a comprehensive review of the literature. Obes Rev. 2020;21(8):e13024. 32207237 10.1111/obr.13024 PMC7379247

[20] Li J, Bai WP, Jiang B, Bai LR, Gu B, Yan SX, et al. Ketogenic diet in women with polycystic ovary syndrome and liver dysfunction who are obese: a randomized, open-label, parallel-group, controlled pilot trial. J Obstet Gynaecol Res. 2021;47(3):1145–52. 33462940 10.1111/jog.14650

[21] Wu W, Zhou Q, Yuan P, Qiao D, Deng S, Cheng H, et al. A novel multiphase modified ketogenic diet: an effective and safe tool for weight loss in Chinese obese patients. Diabetes Metab Syndr Obes. 2022;15:2521–34. 35999869 10.2147/DMSO.S365192 PMC9393022

[22] Emmerson PJ, Wang F, Du Y, Liu Q, Pickard RT, Gonciarz MD, et al. The metabolic effects of GDF15 are mediated by the orphan receptor GFRAL. Nat Med. 2017;23(10):1215–9. 28846098 10.1038/nm.4393

[23] Breit SN, Manandhar R, Zhang HP, Lee-Ng M, Brown DA, Tsai VW. GDF15 enhances body weight and adiposity reduction in obese mice by leveraging the leptin pathway. Cell Metab. 2023;35(8):1341–e13553. 37433299 10.1016/j.cmet.2023.06.009

[24] Siddiqui JA, Pothuraju R, Khan P, Sharma G, Muniyan S, Seshacharyulu P, et al. Pathophysiological role of growth differentiation factor 15 (GDF15) in obesity, cancer, and cachexia. Cytokine Growth Factor Rev. 2022;64:71–83. 34836750 10.1016/j.cytogfr.2021.11.002 PMC8957514

[25] Sigvardsen CM, Richter MM, Engelbeen S, Kleinert M, Richter EA. GDF15 is still a mystery hormone. Trends Endocrinol Metab. Oct 29 2024:S1043-2760(24)00254-6. 10.1016/j.tem.2024.09.002 39472228

[26] Rochette L, Zeller M, Cottin Y, Vergely C. Insights into mechanisms of GDF15 and receptor GFRAL: therapeutic targets. Trends Endocrinol Metab. 2020;31(12):939–51. 33172749 10.1016/j.tem.2020.10.004

[27] Wang D, Townsend LK, DesOrmeaux GJ, Frangos SM, Batchuluun B, Dumont L, et al. GDF15 promotes weight loss by enhancing energy expenditure in muscle. Nature. 2023;619(7968):143–50. 37380764 10.1038/s41586-023-06249-4 PMC10322716

[28] Day EA, Ford RJ, Smith BK, Mohammadi-Shemirani P, Morrow MR, Gutgesell RM, et al. Metformin-induced increases in GDF15 are important for suppressing appetite and promoting weight loss. Nat Metab. 2019;1(12):1202–8. 32694673 10.1038/s42255-019-0146-4

[29] Coll AP, Chen M, Taskar P, Rimmington D, Patel S, Tadross JA, et al. GDF15 mediates the effects of metformin on body weight and energy balance. Nature. 2020;578(7795):444–8. 31875646 10.1038/s41586-019-1911-y PMC7234839

[30] Zhang SY, Bruce K, Danaei Z, Li RJW, Barros DR, Kuah R, et al. Metformin triggers a kidney GDF15-dependent area Postrema axis to regulate food intake and body weight. Cell Metab. 2023;35(5):875–e8865. 37060902 10.1016/j.cmet.2023.03.014 PMC12272050

[31] Aguilar-Recarte D, Barroso E, Zhang M, Rada P, Pizarro-Delgado J, Peña L, et al. A positive feedback loop between AMPK and GDF15 promotes metformin antidiabetic effects. Pharmacol Res. 2023;187:106578. 36435271 10.1016/j.phrs.2022.106578

[32] Søberg S, Sandholt CH, Jespersen NZ, Toft U, Madsen AL, von Holstein-Rathlou S, et al. FGF21 is a sugar-induced hormone associated with sweet intake and preference in humans. Cell Metab. 2017;25(5):1045–e10536. 28467924 10.1016/j.cmet.2017.04.009

[33] Dushay JR, Toschi E, Mitten EK, Fisher FM, Herman MA, Maratos-Flier E. Fructose ingestion acutely stimulates Circulating FGF21 levels in humans. Mol Metab. 2015;4(1):51–7. 25685689 10.1016/j.molmet.2014.09.008 PMC4314524

[34] Lundsgaard AM, Fritzen AM, Sjøberg KA, Myrmel LS, Madsen L, Wojtaszewski JFP, et al. Circulating FGF21 in humans is potently induced by short term overfeeding of carbohydrates. Mol Metab. 2016;6(1):22–9. 28123934 10.1016/j.molmet.2016.11.001 PMC5220397

[35] Fisher FM, Kim M, Doridot L, et al. A critical role for ChREBP-mediated FGF21 secretion in hepatic Fructose metabolism. Mol Metab. 2017;6(1):14–21. 28123933 10.1016/j.molmet.2016.11.008 PMC5220398

[36] Maida A, Zota A, Sjøberg KA, Schumacher J, Sijmonsma TP, Pfenninger A, et al. A liver stress-endocrine nexus promotes metabolic integrity during dietary protein Dilution. J Clin Invest. 2016;126(9):3263–78. 27548521 10.1172/JCI85946 PMC5004939

[37] Richter MM, Thomsen MN, Skytte MJ, Kjeldsen SAS, Samkani A, Frystyk J, et al. Effect of a 6-Week carbohydrate-reduced high-protein diet on levels of FGF21 and GDF15 in people with type 2 diabetes. J Endocr Soc. 2024;8(4):bvae008. 38379856 10.1210/jendso/bvae008 PMC10875725

[38] Ramne S, Duizer L, Nielsen MS, Jørgensen NR, Svenningsen JS, Grarup N, et al. Meal sugar-protein balance determines postprandial FGF21 response in humans. Am J Physiol Endocrinol Metab. 2023;325(5):E491–9. 37729024 10.1152/ajpendo.00241.2023 PMC10874651

[39] Wang Z, Chen T, Wu S, Dong X, Zhang M, Ma G. Impact of the ketogenic diet as a dietary approach on cardiovascular disease risk factors: a meta-analysis of randomized clinical trials. Am J Clin Nutr. 2024;120(2):294–309. 39097343 10.1016/j.ajcnut.2024.04.021

[40] Montefusco L, D’Addio F, Loretelli C, Ben Nasr M, Garziano M, Rossi A, et al. Anti-inflammatory effects of diet and caloric restriction in metabolic syndrome. J Endocrinol Invest. Nov 2021;44(11):2407–15. 10.1007/s40618-021-01547-y PMC8502121 33686615

